# A Mile-Stone on the Road to Registration

**Published:** 1919-01-18

**Authors:** 


### THE MATRONS' AND SISTERS' DEPARTMENT.

### A MILE-STONE ON THE ROAD TO REGISTRATION.

It depends upon nurses whether the conference to be held on Thursday in next week, January 23, at the Royal Society of Medicine, 1 Wimpole Street, W. 1, at 8 P.M., shall be merely a mark-time meeting or such a demonstration of the nurses' determination to get State recognition as shall astound the public at large and awaken the new Minister of Health to a sense of his responsibilities towards the profession.

The movement within the nursing world in favour of obtaining from the State a hall-mark of proficiency has now definitely got beyond the stage of controversy. Like the nation's demand for reconstruction, of which it is a part, it can only accomplish itself through a united will. Old Parliamentary hands have told us how comtemptible they now reckon the endless recriminations and verbal quibbles, the tedious invective and obstruction which in pre-war days attended the passing of any measure for the public good. And they have put on record the amazing change which came over the scene when, in place of acrid debates founded on reluctance to trust the opposite party to carry through their Bills, all parties agreed to facilitate business and get things done to the best of their combined powers.

State Registration for nurses has now reached the stage when the nurses' determination to get it done is the vital factor. There are no outstanding points of principle at issue between the College of Nursing Bill and that promoted by the Central Committee for the State Registration of nurses. Opposition to the College Bill has resolved itself into mere carping at the exact composition of the Provisional Council to be charged with the duties of starting the administration until such time as the representative Nursing Council can get to work, and everyone knows that this is a matter likely in any case to be modified in Committee. The principle of obstruction for personal or party ends has received a knock-out blow in the recent elections. It must no longer be permitted to retard the necessary legislation which nurses demand as their right on the conclusion of war.

The occasion is unique. The nations are conferring that they may make wars to cease and restore tranquillity to a distracted world. Nurses meet in conclave—for what? To demand a higher wage? to press for improved conditions of work? to agitate for material benefits? Not so. They meet to claim from the State a mandate to serve the nation. They have dedicated themselves to the service of humanity, and they ask from the State a seal of their dedication. The rest can wait. Give them but what they ask, the power to make rules for the regulation of their profession, the distinguishing mark of a common sisterhood, fully equipped for their task, and they are confident of accomplishing the rest. They are fully competent to set their own house in order, but before this can be done the State must give them a local habitation and a name.

Let there be no mistake about it. The nation will see that the nurses at this moment have what they want. It is for nurses, then, to show unmistakably that they want State Registration, for once that is demonstrated State Registration they will get. Let them assemble on the 23rd not in clusters of twos and threes, but in parties of twenty and thirty strong. Let them come early, and fill the hall to its utmost capacity long before the hour for the meeting strikes. Let the whole of London know on this occasion that nurses are out to get State Registration, and the effect will be overwhelming and irresistible.

Members of the College, seize your opportunity!

